# A novel mutation in the *ABCD1* gene of a Moroccan patient with X-linked adrenoleukodystrophy: case report

**DOI:** 10.1186/s12883-015-0503-1

**Published:** 2015-11-25

**Authors:** Adnane Karkar, Abdelhamid Barakat, Amina Bakhchane, Houda Fettah, Ilham Slassi, Imen Dorboz, Odile Boespflug-Tanguy, Sellama Nadifi

**Affiliations:** Inserm U1141, Paris Diderot University, Sorbonne Paris Cité, Robert Debré Hospital, Paris, France; Genetics and Molecular Pathology Laboratory, Medical school of Casablanca, Hassan II University, Casablanca, Morocco; Laboratoire de Génétique Moléculaire Humaine, Département de la Recherche Scientifique, Institut Pasteur du Maroc, Casablanca, Morocco; Department of Neuropediatrics, Hassan II University Hospital, 9154 Casablanca, Morocco; Department of Neurology, Hassan II University Hospital, 9154 Casablanca, Morocco; Inserm U1141, Paris Diderot University, Sorbonne Paris Cité, DHU PROTECT, Robert Debré Hospital, Paris, France; Reference Center For Leukodystrophies, Department of Neuropediatrics and Metabolic Diseases, Robert Debré Hospital, AP-HP, Paris, France

**Keywords:** Nonsense mutation, *ABCD1*, ALDP, VLCFAs, X-linked Adrenoleukodystrophy

## Abstract

**Background:**

X-linked adrenoleukodystrophy (X-ALD; OMIM: 300100) is the most common peroxisomal disease caused by mutations in the ATP-binding cassette, sub-family D member 1 gene or *ABCD1* (geneID: 215), the coding gene for the adrenoleukodystrophy protein (ALDP), which is an ATP-binding transport protein associated to an active transport of very long chain fatty acids (VLCFAs). Dysfunction of ALDP induces an accumulation of VLCFAs in all tissues leading to a neurodegenerative disorder that involves the nervous system white matter.

**Case presentation:**

In our case report, magnetic resonance imaging (MRI) as well as the high levels of VLCFAs prompted the diagnosis the X-ALD. Molecular analysis of *ABCD1* gene have shown a pathogenic homozygous nonsense mutation (c.1677C > G; p.(Tyr559*)) in exon 7.

**Conclusion:**

Thus, we identified here a novel mutation in the *ABCD1* gene in a Moroccan patient causing X-linked adrenoleukodystrophy.

## Background

X-linked adrenoleukodystrophy (X-ALD; OMIM: 300100) is the most common peroxisomal disease caused by mutations in the *ABCD1* (OMIM: 300371), the coding gene for the adrenoleukodystrophy protein (ALDP) [1]. ALDP is an ATP-binding transport protein involved in active transport of enzymes or cofactors implicated in very long chain fatty acids (VLCFAs) β-oxidation in peroxisomes. Therefore, a slight dysfunction of ALDP induces an accumulation of VLCFAs in all tissues [2]. This accumulation often leads to a neurodegenerative disorder in the nervous system’s white matter, axons, adrenal cortex, and testes [3] . X-ALD is divided into two main phenotypes: The Child Cerebral ALD (CCALD) and the adrenomyeloneuropathy (AMN). Both of these forms may occur in the same family. However, there is no correlation between mutations in the gene *ABCD1* and the X-ALD phenotype [4]. Epidemiological studies reported that the estimated relative frequency in males with X-ALD is 31–35 % for childhood cerebral ALD and 40–46 % for AMN [5]. Moreover, the total frequency of X-ALD gene carriers is estimated to be 1/16800 for both hemizygous males and heterozygous women [6]. In the present study, a candidate gene approach was used to determine X-ALD related mutation in a Moroccan patient by analyzing the entire coding region of the *ABCD1* gene by direct Sanger sequencing.

## Case presentation

The patient is a 7 years and 5 months old boy who has been adopted at birth with no information about the biological parents, therefore we were unable to realize an adequate family investigation with a pedigree and a molecular analysis for the biological mother. The patient had a normal development until he reached 6 years and 11 months old when he suddenly showed hearing difficulties, a decline in visual acuity and a hyperactivity. He also became violent and failed in school.

**Fig. 1:**
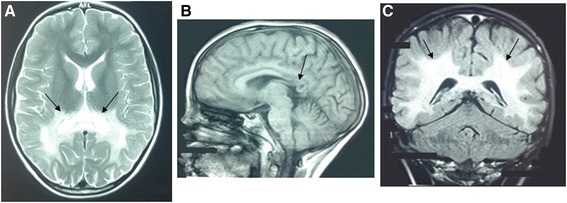
Brain MRI of the patient with high signal intensity of the white matter in axial T2, the arrows show high signal in the corpus callosum (**a**), low signal intensity of the white matter in sagittal T1 (**b**), low signal in the splenium of corpus callosum (arrow B) and diffuse high signal intensity of the white matter in coronal Flair (arrows **c**)

The neurological examination revealed pyramidal syndrome of the lower limbs, walking difficulties and cerebellar ataxia. Besides, MRI images were consistent with active demyelination as usually observed in childhood cerebral ALD. A high bilateral symmetrical signal intensity was detected in T2 and Flair within the pons and midbrain. Furthermore, a low signal intensity was found in T1 and a diffuse high signal intensity of the white matter in T2 and Flair within the parietal-occipital area and the splenium of corpus callosum (Fig. 1).

Visual evoked potential (VEP) and brainstem auditory evoked potential (BAEP) tests have shown that both visual and auditory pathways evoked potential damage. Serum cortisol level reveals that the adrenal glands were not affected. VLCFAs analysis (Table *1*) by liquid chromatography-tandem mass spectrometry (LC-MS/MS) revealed high levels of C24/C22 and C26/C22 ratio in plasma.

**Table 1:** VLCFAs analysis

VLCFAs	Patient	Normal control
C22 mg/L	40.6	1.827 to 22.161
C22 μmol/L	119.364	5.371 to 65.153
C24 mg/L	72.8	1.228 to 18.837
C24 μmol/L	197.288	3.328 to 51.048
C26 mg/L	4.68	<1.03
C26 μmol/L	11.747	<2.585
C24/C22 ratio	1.793	0.665 to 1.008
C26/C22 ratio	0.115	0.009 to 0.069

Genomic DNA was extracted from peripheral blood samples using standard phenol–chloroform extraction method. The DNA concentration and purity were determined using a nanodrop spectrophotometer (NanoVue™-NV - General Eletrics Healthcare Limited, UK). Polymerase chain reaction (PCR) primer pairs of the *ABCD1* coding regions were designed following the NCBI Reference Sequence: NM_000033.3 (Table *2*).

**Table 2:** *ABCD1* gene primers sequences

Exons	Forward 5′ to 3′	Reverse 3′ to 5′	Size
1(first part)	AGCAACAATCCTTCCAGCCA	CACCACGTCCTCCGTCAGA	689
1(second part)	CTGCTACCTTCGTCAACAG	CCCACACCTTTGGCATCAG	593
2	GTGACTAGAGAGGGAGTGG	GGCTTGTCTGAGTGGTAAC	657
3	CATCAGCCTGTGATGTGCTC	TGGGCCTCTTGAAGTGACAG	531
4	GTGAAGAAGGCAGCCTTGG	CCAGAAGCACATGGAGGTC	509
5	AGACTCCCCAGAATGCAGAG	GGCTGAGGCTTGCATATGTG	216
6	CTCTCAAGGCTGGTCAGGAG	CTTCACCACTTCCTGGGCCT	312
7	CGATGTGAGCGTGTGGATG	GGCACCTGGCACTTTAGAC	372
8 and 9	GGAACTGAGCCAAGACCATT	CTGCTGATGACAGCCGCCT	493
10	TGACCCTGTCCCTCTCCTG	GCTGCTGTCTCCTTCATGTG	322

DNA sequence analysis of the ten coding exons of *ABCD1* gene, including the flanking region of each exon (exon-intron boundaries) was performed using conventional Sanger sequencing. DNA samples were first amplified in a final volume of 25 μl containing: 1× reaction buffer, 1× Q-Solution, 200 μM of each dNTP, 2 mM MgCl2, 1 μM of primers, 2.5 U Hotstar Taq polymerase and 50 ng of genomic DNA (Qiagen GmBH, Hilden, Germany). PCR conditions were as follows: denaturation at 95 °C for 15 min; 94 °C for 1 min; 60 °C- for 1 min; and 72 °C for 1 min for 35 cycles followed by a 10 minute final extension at 72 °C. PCR products were electrophoresed on a 1 % agarose gel and visualized with ethidium bromide staining under ultraviolet light to verify their size and quantity. All the PCR products were treated with exonuclease I and shrimp alkaline phosphatase enzymes prior to sequencing according to the following protocol: 37 °C for 40 min and 80 °C for 15 min. The sequencing reaction was performed on the purified products using the BigDye Terminator v 1.1 Standard Kit (Applied Biosystems, Foster City, CA, USA). Electrophoresis of samples was performed on the 3100 ABI Applied Biosystems sequencer (Applied Biosystems, Foster City, CA, USA).

A mutation was identified in the exon 7 of the *ABCD1* gene, with C substituted by G at nucleotide 1677, corresponding to the stop codon at the residue 559.

**Fig. 2:**
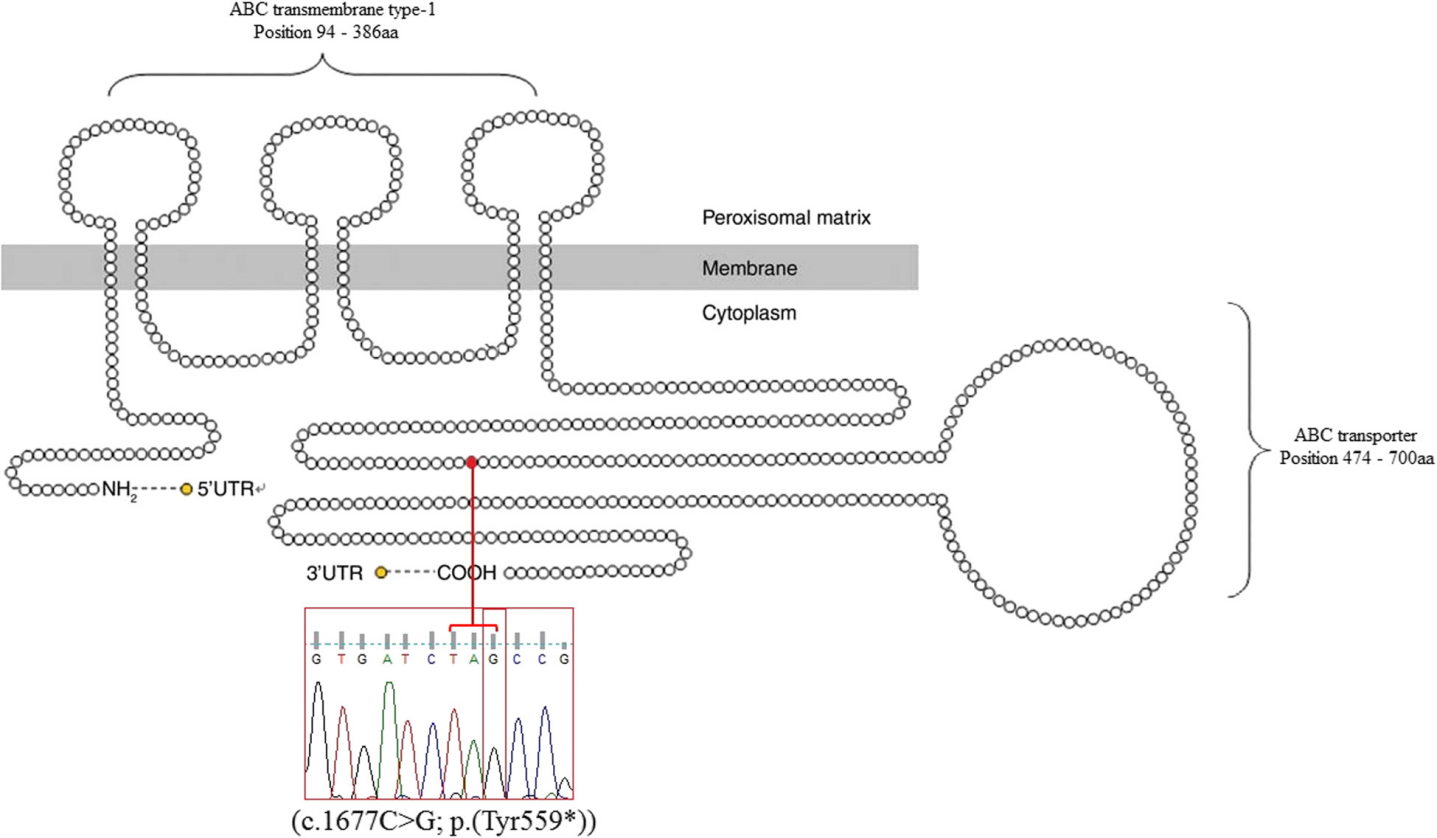
Location of codon 559 in a schematic of ALDP molecular structure [13], and the sequence of the mutation (c.1677C > G; p.(Tyr559*)) in exon 7 of the *ABDC1* gene

The ALDP potentially generated by the appearance of the stop codon exclude a part of exon 7, 8, 9 and 10 coding for the ATP binding domain (Fig. 2).

## Conclusion

The first clinical case of X-linked adrenoleukodystrophy was described by Haberfeld and Spieler in 1910 [7]. Sixty years later, Blaw introduced the name adrenoleukodystrophy relative to adrenal insufficiency correlated with leukodystrophy [8]. In 1981, adrenoleukodystrophy locus was identified at the long arm of the X chromosome [9]. Afterwards, the gene related to the X-ALD (*ABCD1*) was identified using positional cloning strategies in 1993[1].

To date, 1605 mutations have been reported, 703 of them are non-recurrent mutations while 162 others are nonsense and 132 are located in exon 7 (http://www.x-ald.nl). In our study, we identified a novel nonsense mutation in exon 7 (c.1677C > G; p.(Tyr559*)) of a Moroccan patient, there is also another mutation found in the same residue by J. Haasjes & P.A.W. Mooijer in The Netherlands and S.J.S. Steinberg in USA but the nucleotide change is in one position behind our mutation (c.1676A > G; p.(Tyr559Cys)), this mutation is one of the unpublished data in the X-ALD database (http://www.x-ald.nl). No previous studies has investigated the *ABCD1* gene in the Moroccan population. Indeed, only one study reported the same single mutation (c.659 T > C) in three families of Moroccan Jewish descent, probably due to a founder effect [10]. In the North African population, only two other mutations (c.284C > A; p.(Ala95Asp)) and (c.1780 + 2 T > G) of *ABCD1* gene were described in Tunisian patients [11, 12] . All these data lead us to think that the mutations in the *ABCD1* gene are very heterogeneous and their identification in the North African population are very scarce.

Nowadays, Moroccan population has better access to MRI and dosage of VLCFA is requested as soon as the clinical picture is suggestive of X-linked adrenoleukodystrophy. Thus, we expect to have more information about the incidence of the mutations and the frequency of X-ALD within the Moroccan population in the near future.

## Consent

Patient was seen at the Memory Consultation group at the CHU IBN ROCHD Neurology Department in Casablanca, Morocco. The protocol was approved by the human ethics committee of the CHU IBN ROCHD in accordance with the declaration of Helsinki for experiments involving humans and written consent was obtained the guardians prior to the study.

## Abbreviations

X-ALD: X-linked adrenoleukodystrophy
ABCD1: ATP-binding cassette, sub-family D member 1
ALDP: Adrenoleukodystrophy Protein
VLCFAs: Very Long Chain Fatty Acids
MRI: Magnetic Resonance Imaging
CCALD: Child Cerebral ALD
AMN: Adrenomyeloneuropathy
VEP: Visual Evoked Potential
BAEP: Brainstem Auditory Evoked Potential
PCR: Polymerase Chain Reaction
